# An optimization case study for solving a transport robot scheduling problem on quantum-hybrid and quantum-inspired hardware

**DOI:** 10.1038/s41598-023-45668-1

**Published:** 2023-10-31

**Authors:** Dominik Leib, Tobias Seidel, Sven Jäger, Raoul Heese, Caitlin Jones, Abhishek Awasthi, Astrid Niederle, Michael Bortz

**Affiliations:** 1 Fraunhofer ITWM, Optimization Department, 67663 Kaiserslautern, Germany; 2 BASF Digital Solutions GmbH, 67061 Ludwigshafen am Rhein, Germany; 3 BASF SE, 67061 Ludwigshafen am Rhein, Germany

**Keywords:** Mathematics and computing, Applied mathematics, Quantum information, Qubits

## Abstract

We present a comprehensive case study comparing the performance of D-Waves’ quantum-classical hybrid framework, Fujitsu’s quantum-inspired digital annealer, and Gurobi’s state-of-the-art classical solver in solving a transport robot scheduling problem. This problem originates from an industrially relevant real-world scenario. We provide three different models for our problem following different design philosophies. In our benchmark, we focus on the solution quality and end-to-end runtime of different model and solver combinations. We find promising results for the digital annealer and some opportunities for the hybrid quantum annealer in direct comparison with Gurobi. Our study provides insights into the workflow for solving an application-oriented optimization problem with different strategies, and can be useful for evaluating the strengths and weaknesses of different approaches.

## Introduction

Quantum computing (QC) is a field that has witnessed a rapid increase in interest and development over the past few decades since it was theoretically shown that quantum computers can provide an exponential speedup for certain tasks^1–3^. Translating this potential into a practically relevant quantum advantage, however, has proven to be a very challenging endeavor. Nevertheless, the emerging field is considered to have a highly disruptive potential for many domains, for example in machine learning^4^, chemical simulations^5^ and optimization^6^, the domain of this work. Due to the fact that optimization problems are of utmost importance also for industrial applications, we investigated a potential advantage of quantum and quantum-inspired technology for the so-called transport robot scheduling problem (TRSP), a real-world use-case in optimization that is derived from an industrial application of an automatized robot in a high-throughput laboratory. The optimization task is to plan a time-efficient schedule for the robot’s movements as it transports chemical samples between a rack and multiple machines to conduct experiments. This is an NP-hard problem which for certain instances can be challenging to solve using classical computing techniques, and hence is an attractive candidate to search for an advantage with non-classical techniques.

In our study, we compared the solution quality and runtime of different solvers on a large set of instances of the problem. As solvers, we considered D-Wave’s hybrid Leap framework (LBQM) that makes use of the D-Wave quantum annealer^7^, Fujitsu’s digital annealer (FDA)^8^, Fujitsu’s digital annealer hybrid framework (FDAh), as well as the industry-grade Gurobi solver^9^. As a key element of this work, we provide three different models for the TRSP that follow different design philosophies. This is justified by the different ways in which the problem task can be modelled and the inherent differences in the problem formulations that the solvers addressed can accept. LBQM, FDA and FDAh are restricted to a formulation as a quadratic unconstrained binary optimization (QUBO), whereas a mixed integer program (MIP) with integer and float variables can be used by Gurobi, which makes a comparison of multiple formulations meaningful.

The TRSP considered in this paper is a special combination of different scheduling problems that, to our knowledge, has not been considered before. Scheduling problems have been studied intensively for several decades and classical algorithms exist for numerous variants^10,11^. Since most of the industry-relevant scheduling problems are NP-hard, these classical algorithms mainly consist of meta-heuristics or use general-purpose MIP solvers, which basically solve the problem using a branch and bound approach with several additional improvements like cutting planes. In addition to classical algorithmic developments, a considerable amount of research has also been done in hardware-based parallel computing, especially in general purpose computation on graphics processing unit GPGPU parallelization^12,13^. The problem discussed in this work is an extension of the typical job shop scheduling problem JSSP, where the inclusion of a robot adds additional restrictions. More specifically, the studied scheduling problem falls into the category of robotic cell scheduling and automated guided vehicles AGV scheduling problems. Most work on robotic cell scheduling deals with infinite cyclic schedules^14^. This comprises polynomial-time algorithms and hardness results^15^, MIP techniques^16–18^ and heuristic approaches^19^. Many efficiently solvable and hard special cases have been identified^20^ and heuristics have been proposed for some of the hard cases^21^. Those problems differ from our use case in one way or another. The problems considered by the above-cited papers allow, unlike our use case, that the jobs can wait at a machine after their completion before being picked up by the robot. Robotic cell scheduling problems without this possibility have been studied by Ref.^22,23^, whose problems differ from our, among others, in the considered objective function. Our objective function, the total job completion time, has been extensively studied for flow shop scheduling problems without a robot^11,24–26^, the latter of which shows that the no-wait variant is strongly NP-hard on two machines. Apart from the no-wait constraint, the problem considered in our work is characterized by the fact that jobs have to go to the last machine several times. Such settings are known as a re-entrant flow shops, for which Ref.^27^ developed a heuristic algorithm.

We are mainly interested in the performance of non-standard solution approaches using quantum or quantum-inspired solvers in this study. Because these solvers rely on heuristics, benchmarks for real-world applications are a highly relevant research topic. Most quantum optimization approaches fall into two major groups, one for gate-based hardware and one for annealing-based hardware^28^. The majority of gate-based approaches to optimization use parameterized gates to find the ground state of a Hamiltonian related to the cost function of the optimization problem in a quantum-classical hybrid fashion, for example via the quantum approximate optimization algorithm (QAOA)^29,30^.

Approaches based on quantum annealing also seek to find the ground state of a Hamiltonian, but by aiming for an adiabatic change from an initial state that can be easily prepared. In contrast to actual quantum computing devices, other classical software and hardware components are merely *inspired* by quantum computing, for example FDA^31^ and Toshiba’s Simulated Bifurcation (TSB)^32^. Typically, optimization tasks for quantum solvers and the aforementioned quantum-inspired technologies are modeled as QUBO problems^33^. An in-depth analysis of pure QUBO comparison on four quantum and quantum-inspired solvers can be found in Ref.^34^. In their work, the authors compare the solutions of a library of quadratic benchmark problems on the D-Wave quantum annealer, FDA, and TSB against each other.

QC has already been successfully used for optimization in various fields. For example, in Ref.^35^, chemical reaction networks are optimized with quantum computing. In Ref.^36^, it is shown that using the QAOA, it is possible to beat some classical heuristic algorithms on the binary paint shop problem. However, some work has shown that the current circuit model algorithms are not always adequate enough to reach significant convergence required for a good solution^37^. Quantum annealing has proven to offer some advantage against the classical simulated annealing algorithm for a spin-glass problem, using D-Wave hardware^38^, but this is no conclusive evidence. In one of the more recent works on quantum annealing^39^, the authors suggest a nature inspired hybrid quantum algorithm for robot trajectory optimization for PVC sealing in a real industrial setting. In Ref.^40^, the authors present a solution to the maximum independent set (MIS) problem using a Rydberg atom device, along with a claim of a possible super-linear quantum speed-up against classical simulated annealing. Other classical algorithms might still be superior to a quantum approach on current devices^41^. Several works consider scheduling problems^42–44^. In Ref.^45^, an AGV transportation problem using different classical and quantum approaches is studied and Ref.^46^ investigates a nurse scheduling problem with the usage of a quantum annealer.

The remaining manuscript is structured as follows. We provide a detailed description of the TRSP and its mathematical modeling in Sect. "Transport robot scheduling problem". In Sect. "Benchmark setup", we describe the design of our numerical study and list the problem instances and solvers that we use. The results of this study are presented in Sect. "Benchmark results". Finally, we conclude our study in Sect. "Conclusion and outlook". Detailed model descriptions, solver information, further information on the benchmark setup and instance lists are contained in the supplementary material (referenced by a preceding “S” to the label it is referring to).

## Transport robot scheduling problem

In this section, we present a detailed explanation of the TRSP, which is a real-world use case derived from one of BASF’s high-throughput laboratories. This optimization problem is about finding the most time-efficient route of a transport robot tasked with moving chemical samples from one processing machine to another. In the following, we first provide a general description of the problem setup and then present different modeling approaches. These models build the foundation of the subsequent benchmarks.

### Problem description

The laboratory we are modeling consists of a *sample rack* and three different processing machines: a *water mixer*, a *sample shaker* and a *photo booth*. And, finally, the *robot* itself that is tasked with carrying chemical samples from one place to another with the goal to conduct chemical experiments. Only the experimental plan (i. e., how each sample has to be processed in the laboratory) is predefined in advance, but not the specific order of the experiments. Initially, a certain number of samples is stored on the rack. Each of these samples needs to be first taken to the water mixer, then to the sample shaker. Once the sample shaking is completed, one or more photos have to be taken of each sample at the photo booth. Consecutive photos need to be taken after specific (i. e., predefined) time intervals, where the first photo of each sample has to be taken immediately after the shaking process. Finally, each sample has to be brought back to the rack. The processing times for different samples on the same machine can be different as specified by the experimental plan. We assume that each machine can only hold (and process) one single sample at any given time, or remain idle, and the processing steps cannot be interrupted before their completion. It is required that a machine starts processing a sample as soon as the sample is brought by the robot. Moreover, we assume that a sample has to be moved by the robot in-between two processing steps. Hence, a sample has to be lifted from a machine (and the machine is made available) as soon as it finishes processing.

By definition, the robot requires exactly one time unit to move from any place to any other, with or without a sample, and picking up or dropping a sample does not require extra time. Like the machines, the robot can transport only a single sample at any given time or drive empty or remain idle. In particular, it is not possible that the robot places a sample at a machine and picks up another at the same time.

The objective of this scheduling task is to minimize the *sum of sample completion times*, i. e., the sum of the times when the samples arrive at the the rack after their last photo has been taken. The solution of this optimization problem is a sequence of tasks for the robot that yields an efficient laboratory operation.

### Mathematical modeling

In our benchmark, we test three modeling approaches against each other. On the quantum and quantum-inspired side we consider a QUBO formulation, whereas on the classical side we use two MIP formulations. First, a so-called *sequence model* and second, a so-called *time-indexed model*. In the following, we first introduce the common terminology for all modeling approaches. Next, we shortly sketch the main features of each model. For a more detailed description, we refer to Section S1. The motivation for the development of multiple models is to carry out a comparison between the solutions obtained by the most suitable problem encoding for quantum and classical solvers. This ensures that we are comparing the best of both worlds (classical and quantum), and do not restrict ourselves to a model which is more suitable for quantum over classical computing.

#### Common terminology

**Figure 1:**
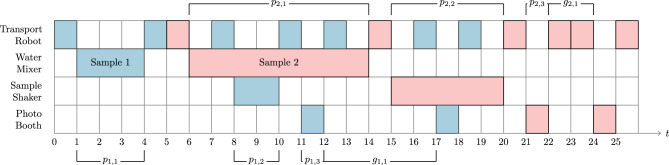
An example Gantt chart of a robot transport scheduling problem with $N=2$ samples and $K=2$ photos.Tasks associated with sample one (two) are colored blue (red). When a sample is processed on one of the machines or carried by the robot in the time-frame $[t,t']$, a bar is drawn from *t* to $t'$ in the respective row in a corresponding color. Empty movements of the robot are not drawn explicitly. For example, at time $t=13$ the robot is at the rack as sample 1 has been brought to the sample rack from $t=12$ to 13. It takes one unit of time for the robot to travel from the rack to the water mixer to pick up sample 2 at $t=14$. From $t=22$ to $t=23$, the sample is brought from the photo booth to the rack and back, which is a consequence of the assumption that a sample has to be moved by the robot in-between two processing steps. The objective value of the depicted schedule is $19+26 = 45$.

The processing machines are addressed by $M_1$ for the water mixer, $M_2$ for the sample shaker and $M_3$ for the photo booth. The scheduling time is discretized into time slots which all have length of one time unit. The transport robot takes one time unit for each operation that is either transportation or empty traversal between the machines and the rack. In this way, each transport robot scheduling problem is uniquely determined by the number of samples to be scheduled $N \ge 1$, the number of photos $K \ge 1$, which agrees for each sample $j \in \{1,\ldots ,N\}$, the processing times $p_{j,1},p_{j,2}, p_{j,3} \in \mathbb {N}_{>0}$ for machines $M_1, M_2$ and $M_3$, which can vary for each sample $j \in \{1,\ldots ,N\}$ and the time gaps $g_{j, k} \in \mathbb {N}_{\ge 2}$ to be kept between consecutive photos *k* and $k+1$ for $k \in \{1,\ldots ,K-1\}$, which also can vary for each sample $j \in \{1,\ldots ,N\}$. As an example, Fig. 1 provides a feasible schedule in form of a Gantt chart to visualize these parameters.

#### QUBO model

A general QUBO reads

1 $$\begin{aligned} \begin{aligned} \min _{x}\quad&x^{\top }\cdot Q \cdot x \\ \text {s.t.}\quad&x \in \{0,1\}^n \end{aligned} \end{aligned}$$

for some matrix $Q \in \mathbb R^{n \times n}$, where *x* represents a vector of *n* binary optimization variables. Two challenging properties of QUBOs must be taken into account in the modeling. Since only binary variables are allowed, this implies that other types of variables must be avoided, i. e. a reformulation into a binary form is necessary. Second, the problem is unconstrained. This restriction can be overcome by using *penalty terms*, which are quadratic functions in the model variables that evaluate to a positive value when the current assignment of values to the variables leads to an infeasible solution. Typically, the penalty terms are designed to yield 0 if the corresponding solution is feasible, so that they do not contribute to the objective values of feasible solutions. More general information about QUBOs and their properties can be found, e. g. , in Ref.^33,47,48^.

Our proposed QUBO model for the TRSP is based on the well-known starting time formulation (see e. g. Ref.^43^) and can be written as

2 $$\begin{aligned} \begin{aligned} \min _{x}\quad&\rho _0 F(x) + \sum _{i=1}^7 \rho _i P_i(x)\\ \text {s.t.}\quad&x\in \{0,1\}^{n}, \end{aligned} \end{aligned}$$

where *F* is the objective function and $P_1, \ldots , P_7$ denote the penalty functions and $\rho _0,\ldots ,\rho _7 \in \mathbb {R}_{>0}$ are tunable parameters that have to be chosen such that the objective and penalty terms are suitably balanced. As in Eq. 1, *n* represents the total number of binary optimization variables. These have a distinct meaning that can be identified with three indices. Specifically,

3 $$\begin{aligned} x_{j,m,t} := {\left\{ \begin{array}{ll} 1, &{}\text {if sample} \;j\; \text {starts processing on machine}\; M_m\; \text {at time}\; t, \\ 0, &{} \text{otherwise}\; \end{array}\right. } \end{aligned}$$

for all $j \in \{1,\ldots ,N\}$, $m \in \{1,2,3\}$ and $t \in \{1,\ldots ,T-1\}$. Here, *T* denotes the time horizon, which is chosen in such a way that there is enough time to schedule all samples sequentially, implying that there is at least one feasible solution. It can be explicitly computed for each instance as described in Section S1.1. In terms of Fig. 1, one has, for example, $x_{1,1,1} = 1$ and $x_{1,2,8} = 1$.

The penalty terms for the QUBO model have to be formulated using the binary optimization variables. This section only provides an example for such a term, a complete description can be found in Section S1.1. Specifically, we consider here the constraint that each sample must access the machines $M_1$ and $M_2$ exactly once, which can be achieved by

4 $$\begin{aligned} P_1 := \sum _{j=1}^N \sum _{m=1}^2 \left[ \left( \sum _{t=1}^{T-1} x_{j,m,t}\right) -1\right] ^2 \;. \end{aligned}$$

This term evaluates to zero if and only if for each pair of sample *j* and machine $M_m$, the variable $x_{j,m,t}$ is 1 for precisely one time slot *t*. Since $P_1$ is bounded below by 0 due to its quadratic nature, each local minimum of $P_1$ is a feasible solution w.r.t. the rule of machine access to $M_1$ and $M_2$. The other penalty terms can be formulated similarly.

Finally, the objective function *F* sums up for each sample the time when the sample arrives at the rack after the entire scheduling process (“sum of sample completion times”). For example, the objective function in the case of Fig. 1 evaluates to 45 time units.

#### MIP models

MIPs have been used since the late 1950s as a tool for solving scheduling problems. It is not possible to model the disjunctive constraints resulting from the discrete ordering decisions only by means of starting time variables. Different types of binary variables have been proposed to achieve this. The main types are position variables $x_{ijk}$ indicating if job *j* is the *k*th job on machine *i*^49^, linear ordering variables $\delta _{ijk}$ deciding if job *j* is processed before job *k* on machine *i*^50^ and time-indexed variables $x_{ijt}$ specifying that job *j* is started (or processed or completed) on machine *i* at time *t*^51,52^. Ref.^53^ compared these three approaches experimentally for a job shop scheduling problem.

Due to the powerful nature of (mixed) integer programming in contrast to the restrictive nature of the QUBO models, we provide two MIP models to be solved using Gurobi, where we follow two state-of-the-art approaches for formulating scheduling problems as MIPs^11^. The first one, in the following named *sequence model*, makes use of continuous start time and binary linear ordering variables. The second model, called the *time-indexed model*, is restricted to a binary formulation comparable to the QUBO model, where we make use of time-indexed variables. The latter provides a model with a natural vicinity to the QUBO formulation whereas the sequence model exploits the features of MIP formulations. In this sense we provide a baseline from two different angles, one for each solution approach.

#### MIP: sequence model

In the sequence model, we model sequences of *events* that affect the behavior of the transport robot with respect to the machines and the photos of a sample. We define the *set of events* as

5 $$\begin{aligned} E := \bigl \{(j, i, a) \mid j \in \{1,\ldots , N\},\, i \in \{1, \ldots , 2 + K\},\, a \in \{0,1\}\bigr \}. \end{aligned}$$

An event $e = (j,i,0)$ represents either that a sample *j* is placed on machine $M_i$ for $i \in \{1,2\}$ or to the $(i-2)$th photo shoot for $i > 2$, an event (*j*, *i*, 1) corresponds to picking it up again. For each event $e \in E$ we define an optimization variable $\tau _e \in \mathbb {R}_{\ge 0}$ to model the time for event *e* to happen. In terms of Fig. 1, we have, for example, $\tau _{(1,1,0)} = 1$ and $\tau _{(1,1,1)}=4$. A simple formulation can be achieved by additionally introducing a binary variable for each pair $e,f \in E$, $e \ne f$ of events that indicates if *e* occurs before *f*. We reduce the size of the model by exploiting the fact that the ordering of some events is fixed or coupled. For example, we do not need a variable that specifies the order in which a given sample is brought to the water mixer and to the sample shaker. This leads to three sets of linear ordering variables that can be found in Section S1.2 , as well as the various constraints to ensure feasibility. The objective function (i. e., the sum of the sample completion times) can be easily expressed using the variables $\tau _e$ corresponding to events when a sample is picked up from the last photo.

#### MIP: time-indexed model

The second constrained model makes use of discrete time-indexed variables similar to the QUBO model from Section S1.1 . In this formulation, we model the behavior of the transport robot by defining certain routes a sample can be transported along, which include those from the rack to all machines and back or movements between subsequent machines. The numbering of the moves is shown in Fig. S1.

As the model name implies, we have, given a discrete time horizon $T \in \mathbb {N}_{>0}$, binary variables to model when each sample takes which route as

6 $$\begin{aligned} y_{j, r,t} := {\left\{ \begin{array}{ll} 1, \text { if sample } j \text { is transported by the robot on route } r \text{ during the time } (t, t+1) \;, \\ 0, \text { otherwise}\; \end{array}\right. } \end{aligned}$$

for all $j \in \{1,\ldots ,N\}$, $r \in \{1,\ldots , 8\}$ and $t \in \{0,\ldots ,T-1\}$. In terms of the Gantt chart from Fig. 1, this would imply $y_{1,1,0} = 1$, $y_{1,2,4} = 1$, $y_{2,1,5} =1$ and so on. The time horizon *T* is defined as for the QUBO model, see Eq. (S2).

The constraints of the model are similar to the penalty terms of the QUBO Model and are listed in Section S1.3. The objective function (i. e., the sum of the sample completion times) is defined in terms of the ancilla optimization variables $z_j$ for $j\in \{1,\dots ,N\}$, that are bounded below by the arrival time of sample *j* at the rack after the schedule has finished.

## Benchmark setup

In the present section, we describe the design of the benchmark. We start with an outline of the considered problem instances that are listed in more detail in Section S2. Subsequently, we describe the three different commercial technologies that we use.

### Instances

To set the stage for our benchmark, we specify 260 test instances of our optimization problem of interest, each defined by a different set of parameters. Specifically, each instance is uniquely determined by the number of samples *N*, the number of photos *K*, the gaps $g_{j,k}$ between subsequent photos *k* and $k+1$ for $k \in \{1, \ldots , K-1\}$ and $j \in \{1, \ldots , N\}$, and, finally, the processing times $p_{j,1}, p_{j,2}, p_{j,3}$ of the water mixer, sample shaker and photo booth, respectively, as explained in Sect. "Mathematical modeling". For the sake of simplicity, the processing time of the photo booth agrees for all samples of the same instance, that is $p_{j,3}:= p_3$ for all $j \in \{1,\ldots ,N\}$.

In Section S2, we describe the algorithm that was used to generate parameter sets for the benchmark instances. Since the resulting instances span a wide range of complexity, we divide the resulting benchmark library into two parts, where each part is defined by the number of binary variables in the corresponding QUBO formulation from Sect. "Mathematical modeling" as explained in Section S1.1 in more detail. The first part, which we call *library of minor instances*, contains all 161 instances that have at least 2071 and at most 8080 binary variables. The second part, which we call *library of major instances*, contains the remaining 99 instances with at least 10822 and at most 22692 binary variables. The reason for that specific division is that 8192 is the maximal amount of variables that can be solved directly on Fujitsu’s digital annealer.

We collect groups of instances (*N*, *K*) that have the same number of samples and photos as shown in Fig. S2, i. e., within those groups the leftover parameters $p_{j,m}$ and $g_{j, k}$ for $j \in \{1,\ldots ,N\}, m \in \{1,2,3\}$ and $k \in \{1,\ldots ,K-1\}$ may vary. These groups can be understood as a collection of “similar” TRSPs in the sense that the complexity of the tasks to be solved is comparable. However, some instances may still be easier or more difficult to solve than others in practice. This grouping approach allows us to consider statistical metrics over several instances when we compare models and solvers. Moreover, it allows us to estimate the scaling behavior of different solution approaches. In Section S2, we list how many instances each group contains.

### Quantum and classical solvers

In our benchmark, we solve the generated instances with a selection of model and solver combinations with the main goal to assess the performance of quantum and quantum-inspired technology. Specifically, we consider three solver candidates:

1. Gurobi: As a baseline, we use the branch and bound algorithm of Gurobi, which is a state-of-the-art mathematical programming solver running on classical hardware^9^. In summary, it relies on an implicit enumeration that allows the original problem to be split into smaller sub-problems using a decision tree. The use of lower bounds derived from linear programming (LP) relaxations allows for a reduction of the search space. Gurobi is an all-purpose solver that can in principle solve the proposed optimization problems to a guaranteed optimality in a deterministic fashion (given sufficient time). In this work we utilized the cloud based service of Gurobi solver, which ran on a Intel(R) Xeon(R) Platinum 8275CL CPU (3.00 GHz with 8 physical cores).
2. D-Wave’s hybrid Leap framework (LBQM): D-Wave provides cloud-based access to their adiabatic quantum computers with over 5000 qubits^7^. By design, their hardware is specifically tailored to solve QUBOs. To this end, the QUBO is encoded in an Hamiltonian such that each optimization variable is represented by one qubit^54^ and the ground state corresponds to the optimal solution. The quantum annealing mechanism aims to find the ground state by performing a suitable time evolution of the quantum system with a subsequent measurement of all qubits to reveal the optimal solution. The D-Wave hardware has only limited connectivity, which means that each qubit can only interact with a certain number of other qubits. This limitation restricts the correlations between optimization variables that can be represented by the Hamiltonian. Finding a suitable representation with these constraints is an NP-hard problem^55^ that has to be solved classically to configure the quantum annealer for a certain problem. In practice, the quantum annealer can typically only be used for QUBOs with much less than 5000 optimization variables. For this reason, D-Wave also provides a hybrid software framework LBQM, which is a black-box algorithm for binary quadratic models (BQMs) that runs on both classical and quantum annealing hardware. It allows larger optimization problems that are too big for the quantum hardware to be handled by presenting only parts of the original problem to the quantum annealer. However, the exact mode of operation of LBQM is not publicly available. In this study, we use only the quantum annealer in a hybrid fashion via LBQM. The quantum machine used in the hybrid framework is the *D-Wave Advantage System 4.1* and the region *na-west-1*. We choose to use a constant number of 1000 samples (or readouts) for all evaluations and use default settings for all parameters.
3. Fujitsu’s digital annealer (FDA) and Fujitsu’s digital annealer hybrid framework FDAh: The digital annealer from Fujitsu can be considered as a quantum-inspired algorithm that runs on dedicated (classical) hardware^31^ and can be accessed using a cloud service. It is based on simulated annealing^56,57^ with two major differences. Firstly, the utilization of an efficient parallel-trial scheme to exploit the parallelization capabilities of the hardware and, secondly, a dynamic escape mechanism to avoid locally optimal solutions. The detailed hardware specifications are confidential. The solver supports QUBOs with up to 8192 variables. In addition, the hybrid solver FDAh is provided to solver bigger problem instances by utilizing both dedicated and classical hardware^8^ similar to D-Wave’s LBQM. In this study, we use both FDA and FDAh. Both solvers require a set of parameters that specify how the annealing is done, which also include the number of repetitions and parallel runs on the chip. The specific parameters we used for FDA and FDAh are provided in Section S3.

In a small pre-study, we excluded a few other solvers; see Section S4. The main scope of the paper is to benchmark the performance of quantum-hybrid and quantum-inspired technologies on the TRSP on a high level against an all-purpose solver with an out-of-the-box performance. In this sense, we also exclude meta-heuristics that are tailor-made to the problem as well.

Each instance can be modelled with each of the three modeling approaches from Sect. "Transport robot scheduling problem". However, not all solvers are applicable to all problem formulations and all instances. The MIP sequence model is solved with Gurobi for all instances. The time-indexed model is solved with Gurobi only for the minor instances. The QUBO model is solved with LBQM and FDA for minor instances. For major instances, the QUBO model is only solved with FDAh.

**Figure 2:**
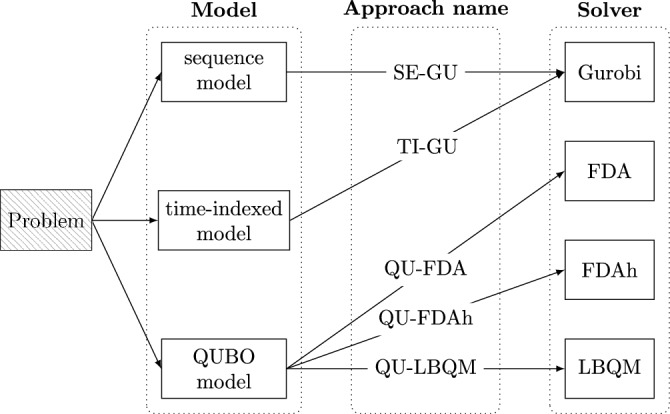
Summary of model (see Section "Mathematical modeling") and solver (see Section "Quantum and classical solvers") combinations for the benchmarks.

We call each valid model and solver combination an *approach* and use a unique name to refer to it. Summarized, we consider Gurobi with the sequence model (SE-GU), Gurobi with the time-indexed model (TI-GU), LBQM with the QUBO model (QU-LBQM), FDA with the QUBO model (QU-FDA) and FDAh with the QUBO model (QU-FDAh). An overview over all approaches is shown in Fig. 2.

For all problems, we prescribe a runtime limit of 3600 seconds for Gurobi. This limit was determined on a heuristic basis, since initial experiments have shown that Gurobi can solve the considered problem instances on this time scale with a practically relevant quality. This time limit exceeds the runtimes of LBQM, FDA and FDAh by far to provide Gurobi enough time to return solutions that are suitable for a relative comparison (see Fig. 4).

Both LBQM and FDA also require a time limit for each run, which scales with the problem size in the QUBO formulation as follows. The time limit for LBQM is set to be $\min \{100, 1.5 \cdot \frac{n}{100}\}$ seconds, where *n* is the number of variables in the QUBO formulation for the minor instances. The runtime of the digital annealer is implicitly set with the *steps* parameter, where each step taken in the annealing process takes a constant amount of time. We set the number of steps to be 1*e*7 for the instances with ${2071} \le n \le {4096}$, 5*e*7 for the ones with ${4096} < n \le {6000}$ and 1*e*8 for the instances with ${6000} < n \le {8080}$ variables in the QUBO formulation. Lastly the major instances computed with the hybrid framework FDAh based on the digital annealer require a time limit as well. For this we distributed the available time of 5 hours to the instances, correspondingly to their number of variables. This computes approximatively as $n \cdot {0.0117}$ seconds where *n* is the number of variables in the QUBO formulation.

The benchmark setup is summarized in Table 1, where we recall the approaches from Fig. 2. The table also contains the values of the QUBO parameters $\rho _0,\ldots , \rho _7$ from Eq. (S16) that were chosen for LBQM, FDA and FDAh, respectively. The choice was made according to previous experiments with smaller problem instances. For this purpose, a typical strategy is to iteratively increase the parameter $\rho _i$ if the corresponding penalty term $P_i$ is non-vanishing. Additionally, one needs to make sure that the parameter $\rho _0$ for the target function is set such that it is not in favor to violate penalty terms and a good optimization is achieved.

Some solutions of the library of minor instances have not been solved to feasibility by LBQM, i. e., the solution vector returned does not translate to a feasible schedule of the TRSP. Those instances can be identified by having an objective value of at least $10^4$, which is the minimum of the penalty parameters chosen for the QUBO model according to Table Table 1. This can be seen as follows: the parameters of the library of minor instances are bounded as $N \le 9$, $K \le 4$, $p_{j,3} \le 3, p_{j,1} \le 8, p_{j,2} \le 4, g_{j,1} \le 5, g_{j,2} \le 12$ and $g_{j,3} \le 24$ for $j=1,\ldots ,N$. Using those upper bounds we compute a maximal time horizon of $T = 648$ time units for those instances. It follows that the sum of sample completion times is bounded above by $9 \cdot 648 = 5832 < 10^4$, i. e., a solution to an instance of the library of minor instances is feasible if and only if it has an objective value below $10^4$. Of course this does neither apply to the library of major instances nor to the solutions of FDA or FDAh as they have lower penalty parameters due to prestudies with the smallest instances. In a general setup a way to identify infeasible solutions is to store the penalty term $\sum _{i=1}^7 P_i(x)$ and evaluate the solution with it. The solution is feasible in this case if and only if the penalty term evaluates to 0 on it.

**Table 1:** Benchmark setup: Summary of problem instances from Section "Instances" and solvers from Section "Quantum and classical solvers" for the optimization problems (or models) from Section "Transport robot scheduling problem".

Property	Minor instances	Major instances
Number of instances	161	99
Number of variables (*n*)	${2071} \text { to } {8080}$	${10822} \text { to } {22692}$
Approach (cf. Fig. 2)	Used for minor instances	Used for major instances
SE-GU	$\checkmark$	$\checkmark$
TI-GU	$\checkmark$	$\times$
QU-LBQM	$\checkmark$	$\times$
QU-FDA	$\checkmark$	$\times$
QU-FDAh	$\times$	$\checkmark$
Approach (cf. Fig. 2)	Minor instance limit	Major instance limit
SE-GU	3600s	3600s
TI-GU	3600s	—
QU-LBQM	$\min \{{100}, {1.5} \cdot \frac{n}{{100}}\} \cdot {1}s$	—
QU-FDA	$${\left\{ \begin{array}{ll} 1 \cdot 10^{7} \text { iterations,} &{}{2071} \le n \le {4096} \\ 5 \cdot 10^{7} \text { iterations,} &{}{4096}< n \le {6000} \\ 1 \cdot 10^{8} \text { iterations,} &{}{6000} < n \le {8080} \end{array}\right. }$$	—
QU-FDAh	—	$n \cdot {0.0117}s$
Solver	QUBO parameters from Eq. (S16)	
LBQM	$\rho _0= {1}, \rho _1= {30000}, \rho _2= \rho _3= \rho _4= \rho _5= \rho _7= {10000}, \rho _6= {15000}$	
FDA	$\rho _0= {1000}, \rho _1= {4000}, \rho _2= \rho _3= {1000}, \rho _4= \rho _5= \rho _6= \rho _7= {1500}$	
FDAh	$\rho _0= {1000}, \rho _1= {2000}, \rho _2= \rho _3= {500}, \rho _4= \rho _5= \rho _6= \rho _7= {750}$	

## Benchmark results

In the current section, we present the results of our previously described benchmark, which is summarized in Table 1. For this purpose, we first show the results for the minor instances and subsequently the results for the major instances.

### Results for Minor Instances

**Figure 3:**
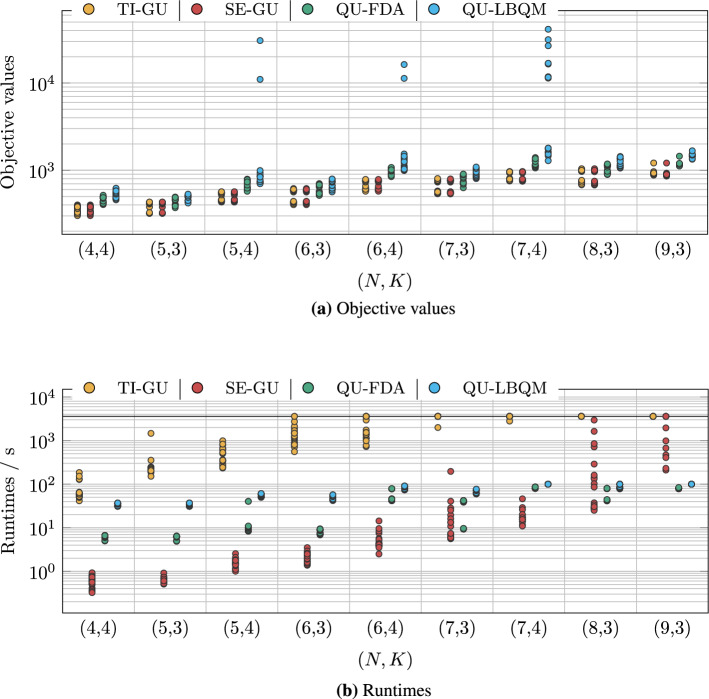
Benchmark results for minor instances as scatter plots. The results are grouped into sets of instances (*N*, *K*) with the same number of samples *N* and photos *K*. A horizontal line marks the upper time limit of 3600s for Gurobi in Fig. 3b. Some instances have not been solved to feasibility by QU-LBQM, as indicated by the peaks above $10^4$ in Fig. 3a. Abbreviations according to Fig. 2.

In Fig. 3, we show the objective values and runtimes of several approaches as scatter plots. All runtimes are end-to-end runtimes, that is, we consider the entire evaluation pipeline, beginning with the submission of the problem to the solver and ending with the return of a solution, including potential network delays. The programmatic construction of the optimization problem for the application programming interface (API) of the solver based on the instance data is not part of the runtime.

From Fig. 3a, we can observe that both the SE-GU and TI-GU solutions reach a better objective value than the solutions from QU-LBQM and QU-FDA. When comparing objective values, it has to be taken into account that the QUBO model objective, Eq. 2, also includes penalty terms, which become positive for infeasible solutions and therefore increase the objective value accordingly. Specifically, we find that only QU-LBQM yields infeasible solutions for some instances, whereas all other approaches yield feasible solutions (SE-GU and TI-GU solutions are by definition always feasible). For our analysis, we include both feasible and infeasible solutions. By performing a Welch t-test^58^, we find that the means of the results from both SE-GU and TI-GU are lower than the means of the QU-FDA and QU-LBQM results with a statistical significance of over $99 \%$, respectively. The same holds for the QU-FDA objective values in comparison to QU-LBQM.

**Figure 4:**
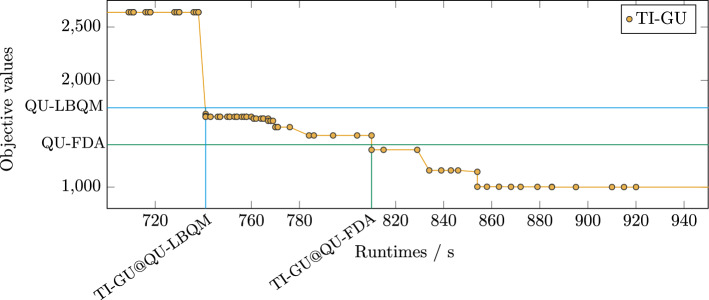
Visualization of the relative runtime of TI-GU w.r.t. QU-LBQM and QU-FDA, denoted by TI-GU@QU-LBQM and TI-GU@QU-FDA, respectively. Here, we consider the example instance (7, 4, 3)(3); see supplementary material. The orange dots (connected by lines for better visualization) mark the resulting objective values of TI-GU at the corresponding time steps. The horizontal upper, blue and lower, green line mark the final objective value of QU-FDA and QU-LBQM, respectively, on the same instance. The blue and green lines intersect with the orange lines at some point. The time coordinate of the next lower TI-GU objective value after this intersection represents the relative runtime of TI-GU w.r.t. the solver, which is marked as a vertical line in the corresponding color. In other words, the relative runtime represents how long TI-GU has to run until it reaches an objective value that is at least as good as the result from QU-LBQM or QU-FDA, respectively.

On the other hand, according to Fig. 3b, the computation time for TI-GU and for some instances of SE-GU exceed the computation time of QU-LBQM and QU-FDA. Since MIP solvers typically spend a lot of time proving that a solution is optimal, we are also interested in the time taken by Gurobi (for both SE-GU and TI-GU) to find solutions of the same quality as those obtained from QU-LBQM or QU-FDA. Hence, we perform an additional analysis of the iterative solver progress of each Gurobi run and look for the earliest computation time at which Gurobi has reached an objective value that is less than or equal to the corresponding objective value returned by the competing solvers for the same instance. We call this earliest computation time the *relative runtime*. Specifically, we consider the relative runtime of TI-GU w.r.t. QU-LBQM (TI-GU@QU-LBQM), the relative runtime of SE-GU w.r.t. QU-LBQM (SE-GU@QU-LBQM), the relative runtime of TI-GU w.r.t. QU-FDA (TI-GU@QU-FDA) and the relative runtime of SE-GU w.r.t. QU-FDA (SE-GU@QU-FDA). In the special case that Gurobi is not able to find an objective value of the desired quality within its limit of 3600 seconds (which only occurs for some major instances), this time limit is used in place of the earliest computation time. Exemplarily, we consider a specific instance to visualize TI-GU@QU-LBQM and TI-GU@QU-FDA in Fig. 4.

**Figure 5:**
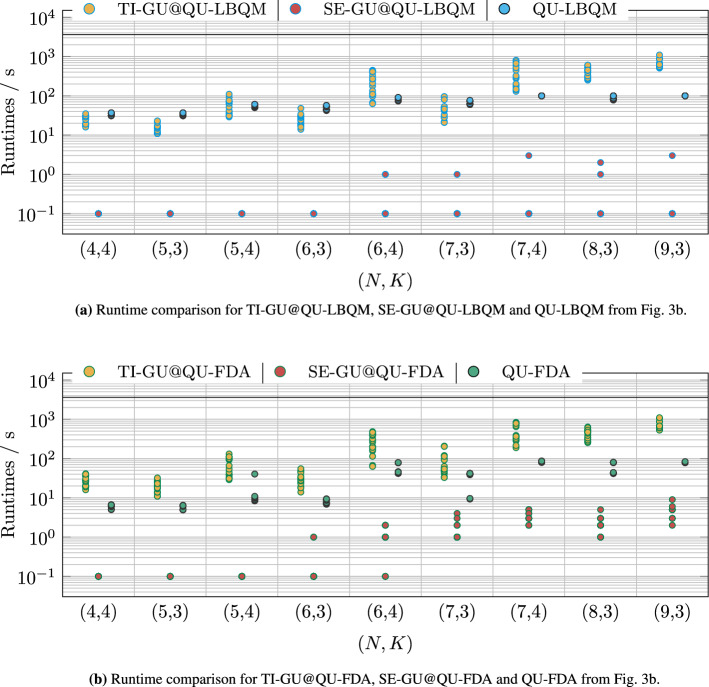
Benchmark results for minor instances as scatter plots. We show the relative runtimes of TI-GU and SE-GU w.r.t. QU-LBQM and QU-FDA, denoted by TI-GU@QU-LBQM, TI-GU@QU-FDA, SE-GU@QU-LBQM and SE-GU@QU-FDA, respectively. The results are grouped into sets of instances (*N*, *K*) in analogy to Fig. 3. See Fig. 4 for an example of the relative runtime computation. Abbreviations according to Fig. 2.

The results of this analysis are shown in Fig. 5. This plot shows that QU-LBQM is not able to compete with SE-GU. All problems from the first 4 out of 9 instance groups have been solved with SE-GU in under 1 second while the remaining instances in less than 10 seconds, whereas the QU-LBQM runtimes range between 50 and 100 seconds. However, LBQM finds a comparable solution faster than TI-GU for most problems with 6 or more samples and remains competitive for smaller problems. A Welch-t test confirms that the mean of TI-GU runtime is larger than the one of QU-LBQM runtime with a significance over $99 \%$.

Furthermore, Fig. 5b shows that QU-FDA is outperformed by SE-GU as well. Analogous to Fig. 5a, the instances in groups (4, 4), (5, 3), (5, 4) and (6, 3) have been solved by SE-GU in 1 second or less. But in contrast to Fig. 5a, the other groups have their median between 1 second and 10 seconds, i. e., which reflects that the target objectives from QU-FDA are lower than those from QU-LBQM (see Fig. 3a). Nonetheless, the time taken for SE-GU to reach the solution quality of QU-FDA is 10 to 100 times smaller. Regarding TI-GU, QU-FDA finds a comparable solution almost always faster with a few exceptions.

### Results for Major Instances

**Figure 6:**
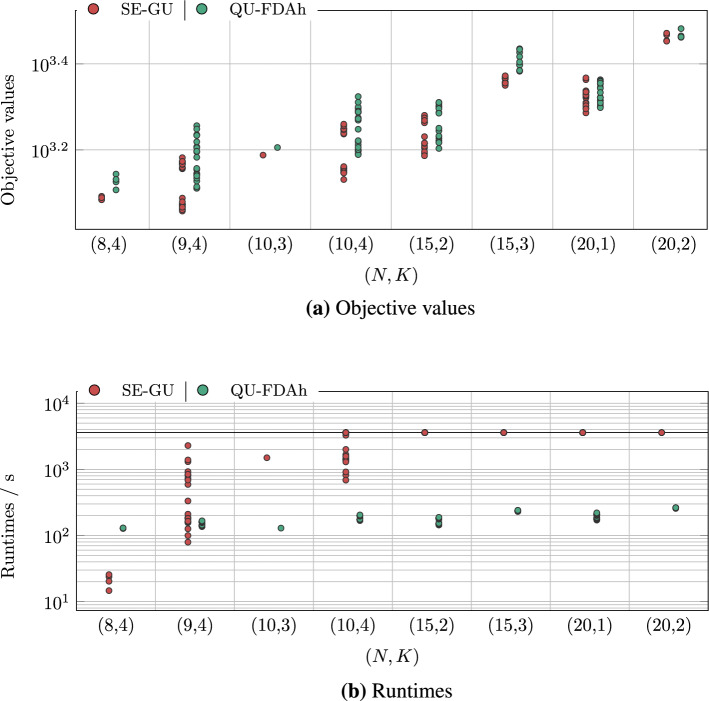
Benchmark results for major instances as scatter plots. The results are grouped into sets of instances (*N*, *K*) as for previous the plots. Abbreviations according to Fig. 2.

The results for major instances are presented in analogy to the results for minor instances from the previous section. In Fig. 6, we show the runtime and the target value of the solvers on the corresponding models as scatter plots.

The objective values of QU-FDAh are worse than the ones of SE-GU with a significance of over $97\%$, but Fig. 6b shows that the runtime of SE-GU increases strictly until it reaches the upper bound for the computation time of 3600 seconds, which happens for ca. 15 samples. On the other hand, the computation time of QU-FDAh ranges between 120 and 300 seconds, where only a slight increase can be seen.

**Figure 7:**
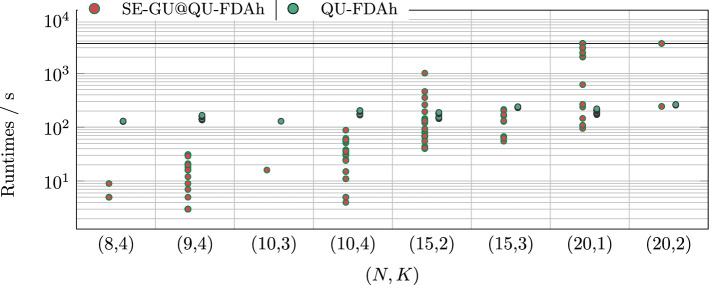
Benchmark results for major instances as scatter plots. We show the relative runtime of of SE-GU w.r.t. QU-FDAh, denoted by SE-GU@QU-FDAh, in analogy to Fig. 5. We also show the runtime of QU-FDAh from Fig. 6b. The results are grouped into sets of instances (*N*, *K*) as for previous plots. Abbreviations according to Fig. 2.

Analogously to Fig. 5b, we evaluate the earliest computation times of SE-GU model to reach objective values equal to or lower than the objective values obtained from QU-FDAh, denoted by the relative runtime of SE-GUw.r.t. QU-FDAh (SE-GU@QU-FDAh). The results are shown in Fig. 7.

In Fig. 7, a strictly increasing computation time can be seen for SE-GU, whereas the QU-FDAh runtime remains almost constant. For the biggest instances with $N=20$ samples, QU-FDAh has a clear advantage with respect to the computation time, whereas it is competitive to SE-GU for the instances with 15 samples. In this sense QU-FDAh finds a solution of comparable quality much faster for problems with 20 samples than SE-GU and the latter was not able to prove optimality for some of the instances with 20 samples. A Welch t-test confirms with a significance of over $99 \%$ that the QU-FDAh mean is lower than the SE-GU@QU-FDAh mean.

## Conclusion and outlook

This paper presents a thorough benchmarking of an industrially relevant use case of combinatorial optimization, the *transport robot scheduling problem *(TRSP) with the goal to achieve a time-optimal robot schedule, as motivated by a BASF high-throughput laboratory. We solve a large set of instances for this optimization problem with varying difficulty using three commercially available solvers: (i) the D-Wave’s hybrid Leap framework, (ii) the quantum-inspired Fujitsu digital annealer and (iii) the classical state-of-the-art solver Gurobi. To this end, we develop several mathematical models: a (QUBO) model for the quantum and digital annealer and two different MIP models for Gurobi, which we call time-indexed and sequence model, respectively. Modeling the same problem in different, solver-specific forms helps us to optimally assess the capabilities of each solver. In total, we compare five different approaches (i. e., model and solver combinations as sketched in Fig. 2): (i) Gurobi with the time-indexed model (TI-GU), (ii) Gurobi with the sequence model (SE-GU), (iii) D-Wave’s hybrid Leap framework (LBQM) with the QUBO model (QU-LBQM), (iv) Fujitsu’s digital annealer (FDA) with the QUBO model (QU-FDA) and (v) Fujitsu’s digital annealer hybrid framework (FDAh) with the QUBO model (QU-FDAh). For our performance study, we separated all problem instances into two groups. First, the *minor instances* with problems less than 10000 binary variables in the QUBO formulation and, second, the *major instances* with problems with more than 10000 and up to 22000 variables. For practical reasons, we only solve the minor instances with SE-GU, TI-GU, QU-LBQM and QU-FDA, whereas the major instances are only solved with SE-GU and QU-FDAh, respectively.

Our benchmark reveals insights both regarding the objective values of the optimization problem (i. e., the sum of sample completion times) as well as the end-to-end runtimes for the considered approaches. Regarding the objective values, we observe for minor instances that SE-GU and TI-GU give similar results, outperforming QU-FDA, which in turn outperforms QU-LBQM (cf. Fig. 3a). For major instances, SE-GU outperforms QU-FDAh (cf. Fig. 6a). Regarding the runtime, we find that for smaller instances TI-GU takes the highest time and SE-GU takes mostly the lowest. Between these two extremes, QU-FDA and QU-LBQM take about the same amount of time (cf. Fig. 3b). However, the runtime of SE-GU significantly increases with increasing instance complexity. This same observation continues for the large instances, for which the runtime of SE-GU is mostly larger than that of QU-FDAh (cf. Fig. 6b).

To get further insights into the relationship between objective value and runtime, we also studied the relative runtime of Gurobi, that is the time that Gurobi took to find an objective value that is at least as good as the final result from another approach. For minor instances, we find that the relative runtimes of SE-GU w.r.t. QU-LBQM and QU-FDA, respectively, are strictly lower than the runtimes of QU-LBQM and QU-FDA, i. e., Gurobi found solutions of comparable quality faster than the quantum and quantum-inspired approaches (cf. Fig. 5a and 5b). This is not surprising since SE-GU tended to find better objectives in shorter time. For major instances, the relative runtimes of SE-GU w.r.t. QU-FDAh increase significantly with increasing instance complexity and clearly exceed the runtime of QU-FDAh for the biggest instances (cf. Fig. 7). Thus, QU-FDAh shows an advantage on some bigger instances. Although the resulting objective values of QU-FDAh were not optimal, the approach shows a clear advantage on some bigger instances when compared to SE-GU on a similar time scale.

Our benchmark spans instances of different scales and therefore allows qualitative estimation of the scaling behavior of different approaches. Specifically, we observe that TI-GU and SE-GU show a runtime that scales exponentially with the instance complexity (as estimated by the number of samples and photos), whereas the runtime of QU-LBQM, QU-FDA and QU-FDAh remains almost constant. The quality of the solutions is not significantly determined by the instance complexity. Further research is needed to investigate and quantify these observations in more detail.

Summarized, no general advantage of the quantum and quantum-inspired solvers was found. However, for certain instances the quantum-inspired hybrid usage of the Fujitsu digital annealer turned out to be a very promising alternative to Gurobi and was clearly superior to the usage of D-Wave’s hybrid Leap framework. Our study is not a conclusive result but rather an application-oriented case study that provides a snapshot of the current technology and leaves room for performance improvements on the modeling as well as the solver side. For example, an improvement of the quantum annealer inside the hybrid framework might be possible with additional problem-specific fine-tuning of the annealing schedule or other hardware-related parameters. Moreover, the recently released constrained quadratic model (CQM) solver from D-Wave also promises to provide much better performance compared to the solver used in this work. Especially in an agile field such as quantum computing, a technology snapshot such as ours can hardly provide any forecasts about future developments. Therefore, in order to preserve an up-to-date assessment, further practical evaluations for real-world use cases will be necessary. The methods and results from this project can serve as a blueprint or at least point of reference for this kind of ongoing research.

## Data Availability

Data of the problem instances and solver configurations are presented within the paper. The code is available upon reasonable request.

## References

[1] Deutsch, D. & Jozsa, R. Rapid solution of problems by quantum computation. *Proc. R. Soc. London. Ser. A: Math. Phys. Sci.* **439**, 553–558, 10.1098/rspa.1992.0167 (1992).

[2] Grover, L. K. A fast quantum mechanical algorithm for database search. In *Proceedings of the twenty-eighth annual ACM symposium on Theory of computing*, 212–219, 10.1145/237814.237866 (1996).

[3] Shor, P.W. Algorithms for quantum computation: discrete logarithms and factoring. In *Proceedings 35th annual symposium on foundations of computer science*, 124–134, 10.1109/SFCS.1994.365700 (IEEE, 1994).

[4] Schuld, M., Sinayskiy, I. & Petruccione, F. An introduction to quantum machine learning. *Contemp. Phys.* **56**, 172–185, 10.48550/arXiv.1409.3097 (2015).

[5] Cao Y (2019). Quantum chemistry in the age of quantum computing. Chem. Rev..

[6] Li Y, Tian M, Liu G, Peng C, Jiao L (2020). Quantum optimization and quantum learning: A survey. IEEE Access.

[7] D-Wave Systems Inc. D-Wave hybrid solver service: An overview. https://www.dwavesys.com/media/4bnpi53x/14-1039a-b_d-wave_hybrid_solver_service_an_overview.pdf (2020). Last accessed 2022-11-08.

[8] Nakayama, H., Koyama, J., Yoneoka, N. & Miyazawa, T. Description: Third generation digital annealer technology. https://www.fujitsu.com/global/documents/about/research/techintro/3rd-g-da_en.pdf (2021). Last accessed 2023-06-13.

[9] Gurobi Optimization, LLC. Gurobi Optimizer Reference Manual (2023).

[10] Brucker, P. *Scheduling Algorithms* (Springer-Verlag, Berlin and Heidelberg, 2007), 5 edn.

[11] Pinedo, M. L. *Scheduling* (Springer International, 2016), 5 edn.

[12] Chakroun I, Melab N, Mezmaz M, Tuyttens D (2013). Combining multi-core and gpu computing for solving combinatorial optimization problems. J. Paral. Distrib. Comput..

[13] Awasthi, A., Läessig, J., Leuschner, J. & Weise, T. GPGPU-based parallel algorithms for scheduling against due date. In *2016 IEEE International Parallel and Distributed Processing Symposium Workshops*, 766–775, 10.1109/IPDPSW.2016.66 (2016).

[14] Dawande, M. W., Geismar, H. N., Sethi, S. P. & Sriskandarajah, C. *Throughput optimization in robotic cells*. No. 101 in ISOR (Springer Science & Business Media, 2007).

[15] Steiner G, Xue Z (2005). Scheduling in reentrant robotic cells: Algorithms and complexity. J. Sched..

[16] Phillips LW, Unger PS (1976). Mathematical programming solution of a hoist scheduling program. AIIE Trans..

[17] Brucker P, Burke EK, Groenemeyer S (2012). A mixed integer programming model for the cyclic job-shop problem with transportation. Discret. Appl. Math..

[18] Feng, J. & Che, A. Robotic cell cyclic scheduling considering cell layout. In *Proceedings of the 32nd Chinese Control Conference*, 2622–2626 (2013).

[19] Liu SQ, Kozan E (2017). A hybrid metaheuristic algorithm to optimise a real-world robotic cell. Comput. Op. Res..

[20] Shabtay D, Arviv K (2016). Optimal robot scheduling to minimize the makespan in a three-machine flow-shop environment with job-independent processing times. Appl. Math. Model..

[21] Stern HI, Vitner G (1990). Scheduling parts in a combined production-transportation work cell. J. Op. Res. Soc..

[22] Agnetis A (2000). Scheduling no-wait robotic cells with two and three machines. Eur. J. Oper. Res..

[23] Agnetis A, Pacciarelli D (2000). Part sequencing in three-machine no-wait robotic cells. Oper. Res. Lett..

[24] Hall NG, Sriskandarajah C (1996). A survey of machine scheduling problems with blocking and no-wait in process. Oper. Res..

[25] Allahverdi A (2016). A survey of scheduling problems with no-wait in process. Eur. J. Oper. Res..

[26] Röck H (1984). Some new results in flow shop scheduling. Z. Oper. Res..

[27] Jing C, Huang W, Tang G (2011). Minimizing total completion time for re-entrant flow shop scheduling problems. Theoret. Comput. Sci..

[28] Alexeev Y (2021). Quantum computer systems for scientific discovery. PRX Quantum.

[29] Farhi, E., Goldstone, J. & Gutmann, S. A quantum approximate optimization algorithm, 10.48550/ARXIV.1411.4028 (2014). 1411.4028.

[30] Blekos, K. *et al.* A review on quantum approximate optimization algorithm and its variants, 10.48550/ARXIV.2306.09198 (2023). 2306.09198.

[31] Aramon, M. *et al.* Physics-inspired optimization for quadratic unconstrained problems using a digital annealer. *Frontiers in Physics* **7**, 10.3389/fphy.2019.00048 (2019).

[32] Tatsumura, K., Dixon, A. R. & Goto, H. FPGA-based simulated bifurcation machine. In *2019 29th International Conference on Field Programmable Logic and Applications (FPL)*, 59–66, 10.1109/FPL.2019.00019 (2019).

[33] Kochenberger G (2014). The unconstrained binary quadratic programming problem: a survey. J. Comb. Optim..

[34] Oshiyama, H. & Ohzeki, M. Benchmark of quantum-inspired heuristic solvers for quadratic unconstrained binary optimization. *Sci. Rep.* **12**, 10.1038/s41598-022-06070-5 (2022). 10.1038/s41598-022-06070-5 PMC8828756 35140264

[35] Mizuno, Y. & Komatsuzaki, T. Finding optimal pathways in chemical reaction networks using ising machines, 10.48550/arXiv.2308.04544 (2023). 2308.04544.

[36] Streif, M., Yarkoni, S., Skolik, A., Neukart, F. & Leib, M. Beating classical heuristics for the binary paint shop problem with the quantum approximate optimization algorithm. *Physical Review A* **104**, 10.1103/physreva.104.012403 (2021).

[37] Awasthi, A. *et al.* Quantum computing techniques for multi-knapsack problems, 10.48550/ARXIV.2301.05750 (2023). 2301.05750.

[38] Raymond J (2023). Hybrid quantum annealing for larger-than-QPU lattice-structured problems. ACM Trans. Quant. Comput..

[39] Schuetz MJ (2022). Optimization of robot-trajectory planning with nature-inspired and hybrid quantum algorithms. Phys. Rev. Appl..

[40] Ebadi S (2022). Quantum optimization of maximum independent set using Rydberg atom arrays. Science.

[41] Albash T, Lidar DA (2018). Demonstration of a scaling advantage for a quantum annealer over simulated annealing. Phys. Rev. X.

[42] Yarkoni, S. *et al.* Multi-car paint shop optimization with quantum annealing. In *2021 IEEE International Conference on Quantum Computing and Engineering (QCE)*, 35–41, 10.1109/QCE52317.2021.00019 (2021). 2109.07876.

[43] Carugno C, Ferrari Dacrema M, Cremonesi P (2022). Evaluating the job shop scheduling problem on a D-wave quantum annealer. Sci. Rep..

[44] Tomasiewicz, D., Pawlik, M., Malawski, M. & Rycerz, K. Foundations for workflow application scheduling on D-Wave system. *In Computational Science-ICCS* **516–530**, 2020. 10.1007/978-3-030-50433-5_40 (Springer) (2020).

[45] Geitz, M., Grozea, C., Steigerwald, W., Stöhr, R. & Wolf, A. Solving the extended job shop scheduling problem with AGVs - classical and quantum approaches. In Schaus, P. (ed.) *Integration of Constraint Programming, Artificial Intelligence, and Operations Research: 19th International Conference, (CPAIOR 2022), Proceedings*, no. 13292 in LNCS, 120-137, 10.1007/978-3-031-08011-1_10 (Springer-Verlag, Berlin and Heidelberg, 2022).

[46] Ikeda, K., Nakamura, Y. & Humble, T. S. Application of quantum annealing to nurse scheduling problem. *Sci. Rep.* **9**, 10.1038/s41598-019-49172-3 (2019). 1904.12139. 10.1038/s41598-019-49172-3 PMC6731278 31492936

[47] Lucas, A. Ising formulations of many NP problems. *Front. Phys.* **2**, 10.3389/fphy.2014.00005 (2014).

[48] Glover, F., Kochenberger, G. & Du, Y. A tutorial on formulating and using QUBO models, 10.48550/ARXIV.1811.11538 (2018). 1811.11538.

[49] Wagner HM (1959). An integer linear-programming model for machine scheduling. Naval Res. Logist. Quart..

[50] Manne AS (1960). On the job-shop scheduling problem. Oper. Res..

[51] Bowman EH (1959). The schedule-sequencing problem. Oper. Res..

[52] Pritsker AAB, Waiters LJ, Wolfe PM (1969). Multiproject scheduling with limited resources: A zero-one programming approach. Manage. Sci..

[53] Ku W-Y, Beck JC (2016). Mixed integer programming models for job shop scheduling: A computational analysis. Comput. Oper. Res..

[54] Zbinden, S., Bärtschi, A., Djidjev, H. & Eidenbenz, S. Embedding algorithms for quantum annealers with chimera and pegasus connection topologies. In Sadayappan, P., Chamberlain, B. L., Juckeland, G. & Ltaief, H. (eds.) *High Performance Computing*, 187–206, 10.1007/978-3-030-50743-5_10 (Springer International Publishing, Cham, 2020).

[55] Lobe, E. & Lutz, A. Minor embedding in broken chimera and pegasus graphs is np-complete, 10.48550/ARXIV.2110.08325 (2022). 2110.08325.

[56] Kirkpatrick S, Gelatt CD, Vecchi MP (1983). Optimization by simulated annealing. Science.

[57] Černý, V. Thermodynamical approach to the traveling salesmanproblem: An efficient simulation algorithm. *J. Opt. Theory Appl.* **45**, 41–51,10.1007/BF00940812 (1985).

[58] Welch BL (1947). The generalization of ‘Student’s’problem when several different population varlances are involved. Biometrika.

